# Incidental Bladder Cancer Detected on Multiparametric Magnetic Resonance Imaging of the Prostate Gland

**DOI:** 10.1155/2015/503154

**Published:** 2015-12-13

**Authors:** Al Sardari, John V. Thomas, Jeffrey W. Nix, Jason A. Pietryga, Rupan Sanyal, Jennifer B. Gordetsky, Soroush Rais-Bahrami

**Affiliations:** ^1^Department of Radiology, University of Alabama at Birmingham, Birmingham, AL 35294, USA; ^2^Department of Urology, University of Alabama at Birmingham, Birmingham, AL 35294, USA; ^3^Department of Pathology, University of Alabama at Birmingham, Birmingham, AL 35294, USA

## Abstract

The increased use of axial imaging in various fields of medicine has led to an increased frequency of incidental findings, specifically incidental cancer lesions. Hence, as the use of multiparametric magnetic resonance imaging (MP-MRI) for prostate cancer detection, staging, and management becomes more widespread, the potential for additional incidental findings in the pelvis increases. Herein, we report the case of a man on active surveillance for low-grade, early-staged prostate cancer who underwent MP-MRI and was incidentally found to have a high-grade bladder cancer lesion.

## 1. Introduction

Prostate cancer is the most common solid organ malignancy in American men and is estimated to account for nearly 28,000 deaths in 2015 [1]. Classically, serum prostate specific antigen (PSA), digital rectal examination, and random systematic transrectal ultrasound (TRUS) guided biopsy are used to diagnose prostate cancer. Multiparametric-MRI (MP-MRI) is a newer modality that can help detect, stage, and guide management of prostate cancer [2–4]. With MP-MRI, functional characteristics of tissues including relative density, neovascularity, and chemical metabolism can be assessed with diffusion weighted imaging, dynamic contrast enhancement, and spectroscopy, respectively.

Bladder cancer is estimated to account for nearly 14,000 cancer-related deaths among men in the United States, with nearly 60,000 new cases diagnosed each year [1]. Although no direct link between prostate cancer and bladder is recognized, a recent study reported that approximately 12% of patients who underwent radical cystectomy for bladder cancer had a history of prostate cancer [5]. The diagnosis of bladder cancer is typically based upon cystoscopic evaluation and histological examination of tissue sampled during cystoscopy prompted by varying degrees of hematuria or lower urinary tract symptoms in patients with recognized risk factors. Approximately 50 to 75% of newly diagnosed cases will be nonmuscle invasive while up to 35% will extend into the detrusor muscle layer but still remain confined to the bladder. Classically, cross-sectional imaging has not been heavily relied upon for local staging but instead used to diagnose regional or metastatic spread [6]. Herein, we report a case of incidental bladder cancer detected via MP-MRI in the setting of prostate cancer active surveillance.

## 2. Case Report

A 65-year-old Caucasian male was found to have low-volume, Gleason score 3 + 3 = 6, early-staged prostate adenocarcinoma found on standard-of-care, extended sextant 12-core TRUS guided prostate biopsy based upon an elevated serum prostate specific antigen level measured at 4.3 ng/mL. He was subsequently referred to our institution for an MP-MRI and possible MRI/US fusion-guided biopsy to evaluate appropriateness of continued active surveillance [4]. Prior to referral he had no imaging performed for prostate cancer staging performed due to the low-volume, low-grade nature of the prostate cancer identified on TRUS guided biopsy.

**Figure 1:**
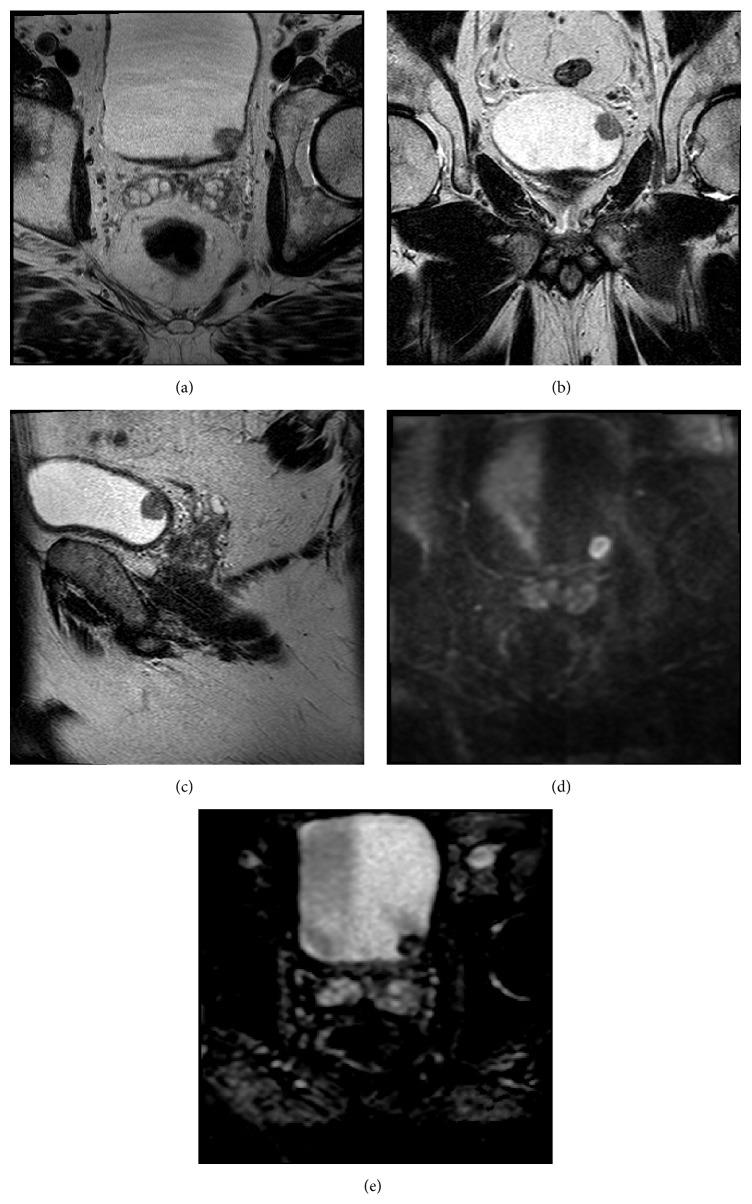
((a), (b), and (c)) Axial, coronal, and sagittal T2 weighted images showing a nodule in left bladder base as a papillary mass of the bladder wall. ((d) and (e)) DWI B2000 and ADC images showing restricted diffusion of bladder mass.

**Figure 2:**
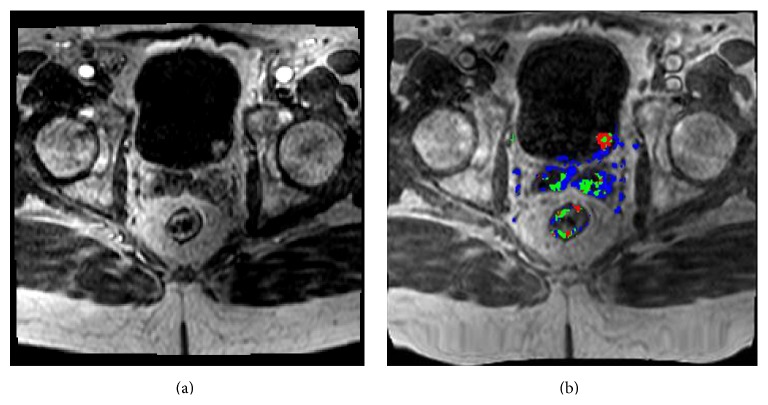
((a) and (b)) Ax T1 dynamic contrast enhanced (DCE) images showing enhancement of the lesion with corresponding increased perfusion on postprocessing color overlay.

**Figure 3:**
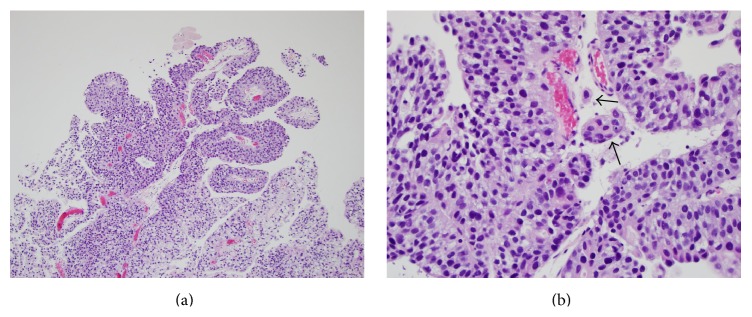
(a) Low power view of high-grade papillary urothelial carcinoma. (b) High power view of invasive high-grade papillary urothelial carcinoma. There is invasion of the lamina propria by tumor cells (arrows).

On the MP-MRI of the pelvis, he was found to have an incidental papillary growth representing a filling defect within the bladder lumen, highly suspicious for a primary bladder tumor (Figures 1(a)–1(e) and 2(a)-2(b)). Urine analysis and culture demonstrated microscopic hematuria and no bacteriuria. He subsequently underwent transurethral resection of the lesion with simultaneous placement of a left ureteral stent given the proximity of the bladder tumor to the left ureteral orifice. Pathology revealed a high-grade papillary urothelial carcinoma with invasion of the lamina propria (Figures 3(a)-3(b)), a synchronous presence of bladder cancer with his prostate cancer. The patient subsequently underwent cystoscopy and reresection to accurately stage the bladder cancer, and it was confirmed to be nonmuscle invasive.

## 3. Discussion

Prostate cancer is one of the leading cancer diagnoses among American men and detection relies on a variety of tests including serum PSA, physical examination including digital rectal examination, prostate biopsy, and noninvasive imaging such as MRI [5]. Current guidelines suggest the greatest benefit for PSA screening is among men aged 55 to 69 years with appropriate pretest evaluation and counseling [6]. Even with these screening and diagnostic tests leading to early detection and treatment, prostate cancer remains one of the leading causes of cancer-related death [1]. Bladder cancer, despite being a much less prevalent malignancy than prostate cancer, accounts for almost half of the number of cancer-related deaths compared to prostate cancer [1]. Bladder cancer does not have any major professional organization recommendations for screening in the general public.

Treatment options for bladder cancer vary based on tumor staging. Early detection and treatment of nonmuscle invasive disease can often avoid invasive, radical surgery. At present, approximately 50–75% of bladder cancers present as nonmuscle invasive tumors. These tumors, clinical Ta and T1 tumors, can be commonly treated and managed with transurethral resection and intravesical therapies. For a clinical T2 tumor, which invades the detrusor muscle layer of the bladder wall, a partial or radical cystectomy is needed for definitive, oncologically sound treatment. Newer developments in noninvasive imaging such as MRI have been reported to help with local staging of bladder cancer though not yet standardized or available as commonplace practice [7].

Multiparametric-MRI (MP-MRI) is a noninvasive imaging modality currently used to assess for prostate cancer. It can be used to locate a tumor and identify spread beyond the prostate. Based upon stage and tumor grade for prostate cancer, management options may include active surveillance, radical prostatectomy, radiation therapy options, and thermal ablative therapies. In addition, data obtained with MR spectroscopy, diffusion weighted, and dynamic contrast enhanced sequences with MP-MRI can provide clinicians with information on tumor characteristics such as aggressiveness [2]. Early studies with 5-year follow-up have shown a negative predictive value near 90% with MP-MRI [8].

Our patient underwent a MP-MRI for prostate cancer surveillance as part of planning for MRI/US fusion-guided biopsy to confirm safe continued active surveillance. Incidentally, a 1.4 cm T2 hypointense/T1 hyperintense nodular, exophytic lesion was identified along the posterior-lateral left bladder wall at the region adjacent to the left ureterovesical junction within the bladder lumen. It demonstrated peripheral diffusion restriction and homogeneous enhancement. There was also an adjacent T1 hyperintense/T2 hypointense linear focus medial to the nodule, which also demonstrated diffusion restriction. There was no evidence of extravesical involvement.

Pathology of the bladder tumor resection demonstrated an invasive high-grade papillary urothelial carcinoma, which invaded the lamina propria. There was no involvement of the muscularis propria. The patient's subsequent reresection of the left bladder hemitrigone two months later demonstrated no evidence of residual carcinoma maintaining the patient as a candidate for further intravesical therapy of his incidental bladder cancer. He continued active surveillance of his early-staged prostate cancer.

Both prostate and bladder cancers are among the most common cancers diagnosed in men with only prostate cancer having major organization guidelines for general public screening at present. There are only a few series reporting the incidence and management for synchronous primary prostate and bladder cancer and hence there are no recognized algorithms for management although in most cases primary management of the bladder cancer is prioritized [9]. MP-MRI is noninvasive imaging modality, now more commonly being employed by clinicians for prostate cancer assessment and active surveillance management. Incidental detection of malignancies such as bladder cancer with MP-MRI can potentially allow for earlier diagnosis and staging of these malignancies, possibly resulting in less invasive treatment options.

## 4. Conclusions

As MP-MRI is more commonly used in the setting of prostate cancer detection and management, recognition of incidental findings on these imaging studies is critical and can serve to expedite early detection and treatment of nonprostate pathologies.

## Abbreviations

MP-MRI: Multiparametric-MRI
MRI: Magnetic resonance imaging
PSA: Prostate specific antigen
TRUS: Transrectal ultrasound
TURBT: Transurethral resection of bladder tumor.
